# Classification of basal stem rot using deep learning: a review of digital data collection and palm disease classification methods

**DOI:** 10.7717/peerj-cs.1325

**Published:** 2023-04-17

**Authors:** Yu Hong Haw, Khin Wee Lai, Joon Huang Chuah, Siti Khairunniza Bejo, Nur Azuan Husin, Yan Chai Hum, Por Lip Yee, Clarence Augustine T. H. Tee, Xin Ye, Xiang Wu

**Affiliations:** 1 Department of Biomedical Engineering, Universiti Malaya, Kuala Lumpur, Malaysia; 2 Department of Electrical Engineering, Universiti Malaya, Kuala Lumpur, Malaysia; 3 Department of Biological and Agricultural Engineering, Universiti Putra Malaysia, Serdang, Selangor, Malaysia; 4 Department of Mechatronics and Biomedical Engineering, Universiti Tunku Abdul Rahman, Bandar Sungai Long, Cheras, Kajang, Selangor, Malaysia; 5 Department of Computer System and Technology, Universiti Malaya, Kuala Lumpur, Malaysia; 6 College of Physics and Electrical Information Engineering, Zhejiang Normal University, Zhejiang, China; 7 YLZ Eaccessy Information Technology Co., Ltd, Xiamen, China; 8 School of Medical Information and Engineering, Xuzhou Medical University, Xuzhou, China

**Keywords:** Basal stem rot, *Ganoderma boninense*, Oil palm, Remote sensors, Deep learning

## Abstract

Oil palm is a key agricultural resource in Malaysia. However, palm disease, most prominently basal stem rot caused at least RM 255 million of annual economic loss. Basal stem rot is caused by a fungus known as *Ganoderma boninense*. An infected tree shows few symptoms during early stage of infection, while potentially suffers an 80% lifetime yield loss and the tree may be dead within 2 years. Early detection of basal stem rot is crucial since disease control efforts can be done. Laboratory BSR detection methods are effective, but the methods have accuracy, biosafety, and cost concerns. This review article consists of scientific articles related to the oil palm tree disease, basal stem rot, *Ganoderma Boninense*, remote sensors and deep learning that are listed in the Web of Science since year 2012. About 110 scientific articles were found that is related to the index terms mentioned and 60 research articles were found to be related to the objective of this research thus included in this review article. From the review, it was found that the potential use of deep learning methods were rarely explored. Some research showed unsatisfactory results due to limitations on dataset. However, based on studies related to other plant diseases, deep learning in combination with data augmentation techniques showed great potentials, showing remarkable detection accuracy. Therefore, the feasibility of analyzing oil palm remote sensor data using deep learning models together with data augmentation techniques should be studied. On a commercial scale, deep learning used together with remote sensors and unmanned aerial vehicle technologies showed great potential in the detection of basal stem rot disease.

## Introduction

Oil palm (*Elaeis guineensis Jacq*.) plays a crucial role in the agricultural industry in developing nations (Siddiqui et al., 2021). Palm oil contributes to one third of the world’s vegetable and fat supply (Bharudin et al., 2022), which shows that the crop is of vital importance to support the world’s population and food need (Nurazah et al., 2021). In a global scale, 74% of the global palm oil is consumed as food items and 24% of the oil is used for industrial purposes.

Globally, oil palm has a huge global market which is valued at USD 65.73 billion in year 2015. The industry was expected to expand to above USD 92 billion in year 2021. Out of the global oil palm crop supply, 85% or more of the supply comes from Indonesia and Malaysia (Bentivoglio, Finco & Bucci, 2018). With the rise of concern over climate change, demand for a renewable source of oil such as palm oil is expected to grow (Siddiqui et al., 2021).

Being the global market leader in oil palm production, Indonesia and Malaysia have seen a yield increase of 4% annually from year 1998 to 2008 (Siddiqui et al., 2021). However, a decline in growth has been observed in both Malaysia and Indonesia since year 2009, indicating several factors have negatively impacted the growth rate of the oil palm crop (Siddiqui et al., 2021).

Alam, Er & Begum (2015) described that several factors have impeded the growth of the industry, *i.e*., labour shortage, competition with neighboring countries, ageing oil palm tree, biodiversity, plant disease and infections and other factors. The plant disease mentioned that has severely impacted the growth of the industry is called the basal stem rot (BSR). BSR has directly caused a significant reduction in the yearly harvest of the oil palm (Rani et al., 2020).

The following three sections review in details about the BSR and *Ganoderma boninense (G. boninense)*, the economic impact should the spread of BSR persist, and how BSR can be controlled and managed.

### BSR and *G. boninense*

BSR is a major oil palm disease that can be found in countries that has a strong dependence on the oil palm industry, such as Malaysia and Indonesia (Nusaibah et al., 2016). BSR is a disease that is caused by the white rot fungus, *i.e*., *Ganoderma* (Sujarit et al., 2020). Of all the species of *Ganoderma* that cause BSR in oil palm tree, *G. boninense* is the most frequently seen *Ganoderma* fungi that causes the BSR disease (Bharudin et al., 2022).

*Ganoderma boninense* is a soil-borne pathogen. The fungus is capable of spreading and colonizing through the roots, shoots, stems and rotting stem tissues (Sujarit et al., 2020). *Ganoderma boninense* is classified as a necrotrophic fungus, which is a type of fungus that is inconspicuous during the early stage of infection while the fungus degrades the cell wall of the host to obtain nutrients by producing a wall-degrading enzyme (Alexander et al., 2017). The infection of *G. Boninense* leads to wood decay at the basal part of the tree, causing a whitish colour with a fibrous texture (Nusaibah et al., 2016; Sujarit et al., 2020).

**Figure 1:**
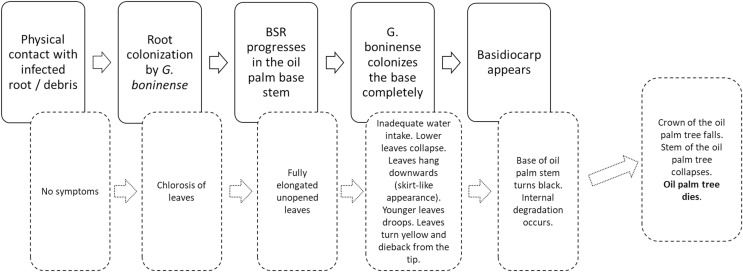
Schematic representation of BSR disease progression (solid line) and the respective symptoms in oil palm trees (dashed lines) (Siddiqui et al., 2021).

According to Nurazah et al. (2021), *G. Boninense* spread directly to healthy host plants either by root-to-root contact, basidio-spores or free secondary inoculum in soil. During the early stage of infection, the host typically shows no symptom. As the root becomes colonized by *G. Boninense*, oil palm tree leaves will begin to show Cholorosis which is a condition where leaves produce insufficient chlorophyll. As the infection worsens, the BSR symptoms begin to show at the shoot of the host. The new leaves will become fully elongated but remain unopened which they are called spears. A complete colonization by *G. Boninense* causes a restricted water uptake by the host which causes the lower leaves to collapse and hanging downwards, which gives a skirt like appearance. The younger leaves will droop, turn yellow and die back from the tip. At the base of the stem, the base turns black and there is severe degradation within the stem. Basidiocarps begin to appear at the base of the plant during this stage. At the end of the disease progression, the crown of the oil palm fall off and the foundation of the tree becomes fractured. During the late stage the tree collapses, which marks the death of the tree (Siddiqui et al., 2021; Sujarit et al., 2020). Figure 1 shows the BSR disease progression (solid line) and the respective symptoms in oil palm trees (dashed lines) (Siddiqui et al., 2021).

### Economic impact of basal stem rot

It was estimated that when an oil palm tree reaches half of the tree’s average economic life, BSR can cause four out of five trees to die if no control measure was taken. A total of 43.32% yield reduction was also expected as the fruit branches experience weight loss due to BSR (Nurazah et al., 2021). From infection to the eventual death of oil palm, an oil palm tree will die within 2 to 3 years (Bharudin et al., 2022; Nurazah et al., 2021).

The significant reduction in yield and lifespan of oil palm tree caused by BSR necessitates the infection control and replanting, which cause a significant economic loss to Malaysia. Infection rate of BSR was estimated at 1.5% in year 1995 and the rate of infection increased by nearly 400% after 22 years (infection rate is estimated at 7.4% in year 2017) (Bharudin et al., 2022). It was estimated that by year 2020, 60 million oil palm trees could be infected by *G. Boninense* in Malaysia (Midot et al., 2019).

As a result, Rani et al. (2020) estimated that BSR causes an RM 225 million economic loss in Malaysia *per annum*. Bharudin et al. (2022) estimated that the economic loss is up to USD 500 million (about RM 2.2 billion) *per annum*. It was also estimated that BSR can potentially destroy 860,610 hectares of oil palm plantations in Malaysia by year 2040, which may be a major concern to the sustainability of the crop to meet the crop’s global demand (Olaniyi & Szulczyk, 2020).

Currently, BSR is still considered a containable disease at the current level. However, based on the projected climate change done by Paterson (2019), if climate change occurs as projected, oil palm tree may face an increase in virulence of *G. Boninense* and also a decrease in suitable climate conditions for palm plantation. Oil palm in Sumatra specially will be critically impacted by the lack of suitable climate from year 2050 onwards, leading to a projected significant increase in BSR.

### Management of basal stem rot

Physical control of BSR is the primary method to control the BSR disease. The physical method includes sanitization methods such as prevention of contact with infected trees. Methods of physical control to minimize the spread of BSR include clean clearing, isolation trenching, ploughing, harrowing, burning, fallowing and windrowing (Hushiarian, Yusof & Dutse, 2013). However these procedures are often found to be expensive while not being fully effective in preventing the infection of healthy oil palm. Burning of infected plant was also done to control the spread of BSR, however this method was outlawed by Malaysian government under the environmental quality act (EQA 1974) (Siddiqui et al., 2021; Ho et al., 2016).

Chemical control and treatment requires the use of fungicides. However, the chemical treatment method to field control BSR was often unsuccessful (Sujarit et al., 2020). There is also growing interest in the use of low molecular weight phenolic compounds as part of the chemical treatment. However, the use of phenolic compounds in the field need to be carefully researched as the impact towards the environment is concerning as the compounds are often toxic (Siddiqui et al., 2021).

Manipulating the nutrient intake by the oil palm is also a method that is currently investigated. The nutrient intake manipulation is done by modifying the fertilizers used on the oil palm seedlings. Example given was NPK fertilizers used in a study done by Sahebi et al. (2018). The results appears promising however the solution is not adequate in controlling the BSR disease (Siddiqui et al., 2021).

Biocontrol was tried in many research however the method shows little successes and practicality due to the lack of effectiveness in field (Siddiqui et al., 2021). The method is also susceptible to hostile environment conditions. Example provided by Nurazah et al. (2021) was the use of *Tri-choderma* spp. and chemical fungicides as biocontrol agents, but both agents failed to significantly control the BSR disease.

Rapid biodegradation of diseased and fallen tree is a potential BSR disease control measure. Once an infected oil palm tree die and fall because of BSR, the trunk and debris of the dead oil palm tree remain a good source of nutrient for the proliferation of *Ganoderma*. The degradation of a dead oil palm takes typically 10 to 24 months. Several other fungi were tested in other studies to increase the rate of deterioration for the dead oil palm as a method to quicken biodegradation of fallen oil palm tree to control the spread of BSR. Although promising, the safety of this method towards human and other environmental microbiome should be thoroughly studied before the application on a commercial scale (Siddiqui et al., 2021).

Genetic resistant material was considered to be one of the methods to control BSR disease. The progress of the use of resistant planting materials have been impeded due to the lack of plant resistance source. Screening for the resistance source is also inefficient. Through multiple studies, a partial resistance source has been found and a screening technique was developed, however expanding the use of these traits require backcross breeding to advanced breeding parents that needs at least five to six generations (about 30 to 40 years). This is due to the resistant materials being derived from semi-wild genotype. Development of an oil palm tree that is fully resistant to BSR is still challenging as the genes that are related to deference are still not isolated (Siddiqui et al., 2021).

Tisné et al. (2017) tested on a QTL mapping approach. This approach is found to be flexible and efficient. The approach is capable of generating unique and valuable information that can help with the selection of tree varieties that show resistance to *Ganoderma* disease. From the said study, four *Ganoderma* resistance loci were characterized, where two of the said loci are responsible for the control of the manifestation of the first *Ganoderma* symptoms, while the other two loci are responsible for the process of death of palm trees. For ongoing breeding programs, characterization of favorable genetic variations can be done among a major gene pool. This transgenic method is promising but immediate commercial adaptation is unlikely (Siddiqui et al., 2021). While waiting for the realization of *Ganoderma* resistant palms on a commercial scale, deboling, *i.e*., removal of diseased palms is still the most important BSR disease control measure at this moment (Hushiarian, Yusof & Dutse, 2013).

Since there are no effective treatment for BSR once the infection begins, the most effective method to limit the spread of BSR is to detect and isolate infected trees during early stages of infection. Early detection of BSR remains a difficult challenge as the oil palm will appear symptomless during early stages of *G. Boninense* infection (Siddiqui et al., 2021). Therefore, detection method that is quantitative, sensitive and selective need to be developed to detect BSR at the early phase. The solution should also be affordable, having high tolerance towards hostile environmental conditions and not requiring specialized equipment or training (Rani et al., 2020).

The review in chapter 1 shows that it is possible to control the spread of BSR to prevent further losses in yield and production. In the following chapter, a review was done on the latest research that is related to the detection of BSR infection. The purpose of the following review is to identify which methods were tried and also to discover knowledge gaps that can be further studied to improve the detection of BSR infection at the early phase, in hope for a more accurate detection and potentially used on a commercial scale. This review is intended for researchers who are working on the improvement of existing BSR detection methods, or researchers that intend to utilize machine learning models to classify and determine plant diseases.

## Literature review

### Survey methodology

In this literature review, scientific articles since year 2012 that are listed in the Web of Science that are related to the oil palm tree disease, basal stem rot, *Ganoderma Boninense*, remote sensors and deep learning were thoroughly reviewed and summarized below. The purpose of this review is to give a full insight to the latest technology used in the detection and classification of BSR. About 110 scientific articles were found to be related to the index terms mentioned above, and around 60 scientific articles were found to be related to the objective of this review.

The review article also included research articles that are related to other plant diseases and the detection of those plant diseases using machine learning. The purpose of reviewing other plant disease is to determine if it is possible for machine learning models to identify diseased plants from the sample.

### Manual and laboratory BSR detection method

Before the introduction of technology, the manual method was often the only method to detect BSR infection (Tee et al., 2021). The manual detection method is still done today, primarily by having workers perform BSR inspection visually based on the external symptoms that are visible. Typically the signs of BSR can be found on fruits, leaves, stems and roots of the tree (Ibrahim et al., 2020). However, at early stage of BSR infection, the symptom is often not detectable visually, thus making BSR detection *via* the manual method exceedingly not viable (Siddiqui et al., 2021). Besides, visual detection method is often heavily dependent on the expertise and experience of the observers, and the skillset is often not transferable, or at best takes long period of time to be passed down.

This manual method is also heavily dependent on manpower, which is prone to human error (Tee et al., 2021). Alam, Er & Begum (2015) also mentioned a labor shortage problem that can worsen the rate of BSR detection. The recent land labor ratio estimated that one worker covers about 10.9 ha plantation. With these consideration in mind, involvement of technology is a must to provide a more efficient solution towards BSR detection.

Lab based methods were extensively reviewed by Tee et al. (2021). Three methods were discussed by the authors, which are ganoderma selection medium (GSM), polymerase chain reaction (PCR) and enzyme-linked immunosorbent assay polyclonal antibodies (ELISA-PABS) (Tee et al., 2021).

GSM is not a recommended method for commercial scale use as one of the reagents, pentachloronitrobenzene (PNCB), is considered hazardous and toxic in certain countries, such as the United States and Indonesia. There were also concerns over the accuracy of GSM (Tee et al., 2021).

PCR is a technology that amplifies the DNA target from a DNA mixture. PCR is often considered the gold standard of lab test due to the test’s accuracy and sensitivity. However, PCR testing requires expensive infrastructure, highly skilled laboratory personnel, complex protocols and high reagent costs. PCR also does not support on site analysis as the samples have to be transported to a lab environment in order to be tested, which makes the test time consuming. Siddiqui et al. (2021) and Hushiarian, Yusof & Dutse (2013) both mentioned that PCR test is also prone to contamination. Having PCR as a large-scale monitoring solution is also not viable as PCR test lack of ability to classify the status of infection (Tee et al., 2021; Martinelli et al., 2014). Therefore, using PCR test to intensely detect BSR disease on a large scale is not the best option.

ELISA-PABS is an immunology assay that is capable of rapidly quantifying a particular protein molecule using certain highly specific antibodies found in a protein mixture. ELISA-PABS offer a simpler approach and require less equipment compared to PCR tests. ELISA-PABS also showed better detection performance in terms of accuracy and sensitivity when the test is compared against GSM (Madihah, Idris & Rafidah, 2014). Despite the positive results compared to GSM and PCR, ELISA-PABS is still not suitable when it is considered for large scale monitoring and detection since the test is still sophisticated and time-consuming (Tee et al., 2021).

To summarize, manual method and lab methods are both not suitable for extensive use towards monitoring, detection and classification of BSR disease. As sensor, imaging and analytical technologies improve, digital data collection together with remote classification method such as machine learning should be considered for the early detection of BSR disease. The digital detection and classification methods have great potential to be used for large scale monitoring due to the reduction of labor and hours compared to manual detection and lab methods (Tee et al., 2021).

### Digital data collection and remote BSR classification methods

Several methods of digital data collection will be reviewed under the section below, together with the classification algorithm attempted by several authors and the author’s recommendation as applicable.

#### Terrestrial laser scanning

Terrestrial laser scanning (TLS) is known as a ground-based light detection and ranging (LiDAR). TLS is a technology where an active remote sensor captures the image by means of laser light. The laser light was shot in a pulsed manner and the reflected pulses from the object will then be captured by a sensor to directly represent the external structures of the object. The signal is then used to profile the targeted object (Tee et al., 2021).

**Figure 2:**
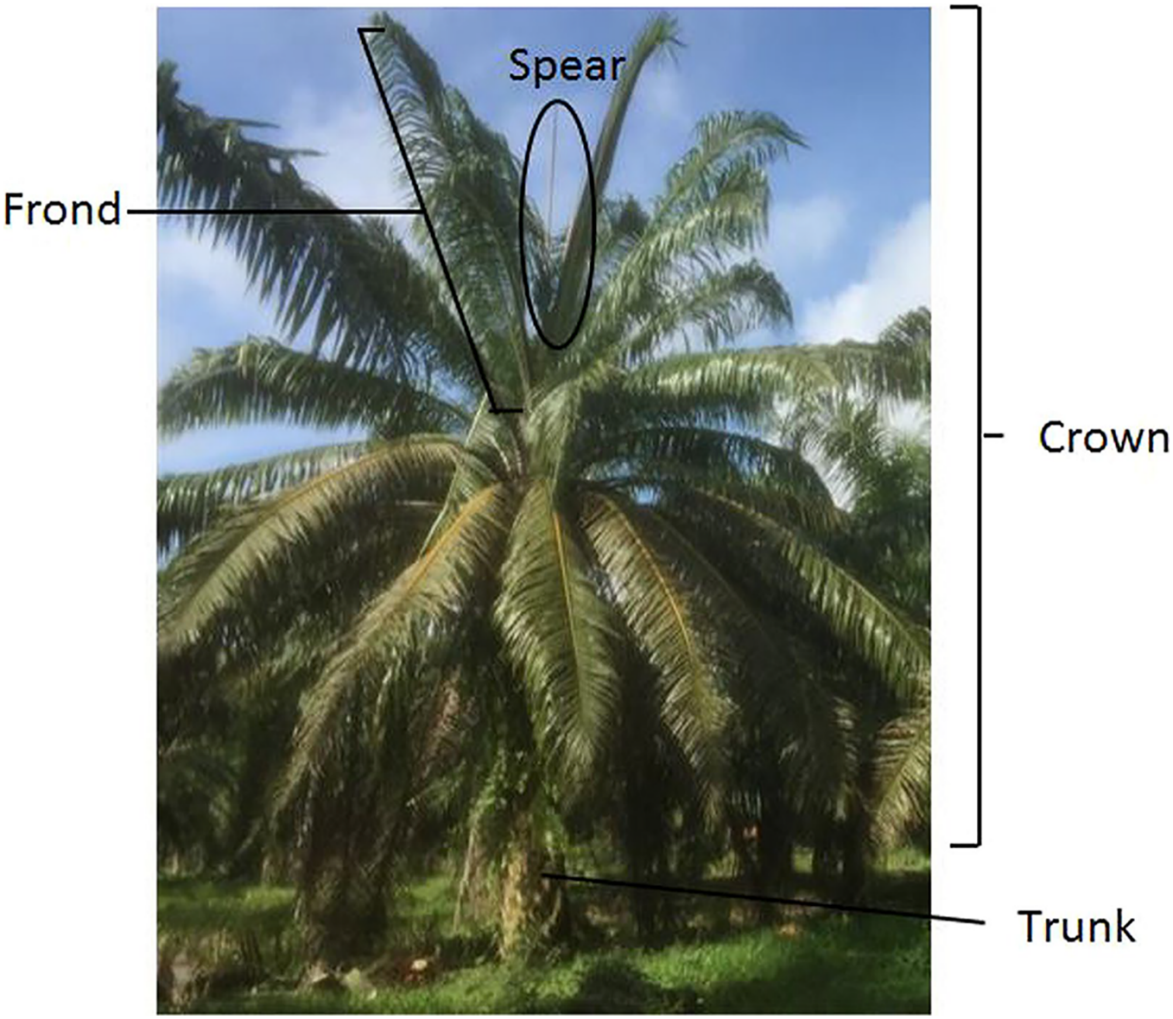
Oil palm morphology, illustrated by Husin et al. (2020c).

The potential use of TLS to classify BSR infection was first investigated by Khairunniza-Bejo & Vong (2014). Faro laser scanning focus 3D (FARO Technologies, Inc., Lake Mary, FL, USA) was used to acquire, pre-process and then analyze two regions of interest on an oil palm tree, *i.e*., the trunk and canopy. The scanned data of the trunk was sliced horizontally to find the significant difference that can distinguish infected trees from the healthy ones. Analysis done on the canopy was primarily focused on canopy size and number of unopened new fronds. Linear correlation between tree properties namely perimeter and area of trunk *versus* the health level were analysed using Pearson correlation coefficient. The results demonstrated at 150 cm height of the palm, the correlation between area and perimeter of trunk *vs* BSR severity is significant with coefficient of determination, R^2^ = 0.8814 and 0.7312 respectively. In short, the smaller the area and perimeter of the trunk, the higher the severity of BSR. The canopy top view also demonstrated a significant correlation with the health level for oil palm with a Pearson correlation value of −0.806 where the higher the BSR severity, the smaller the oil palm’s canopy cover. Figure 2 shows the oil palm morphology as illustrated by Husin et al. (2020c).

FARO Laser Focus^3D^ X120 (FARO Technologies, Inc., Lake Mary, FL, USA) was also used by Husin et al. (2020c) to classify the BSR disease using canopy stratification. Typically for an oil palm that is infected by BSR, the “skirting” effect on the canopy is the most recognizable BSR symptom. 3D images of the tree were constructed using laser point data. The tree canopy was then stratified horizontally. The data were then classified using six classification models, where linear model with a combination of frond number, frond angle and canopy strata at 200 cm from the top showed the highest accuracy for healthy tree classification (100%) while showing a respectable high accuracy for healthy-unhealthy classification (Husin et al., 2020c).

In another study, Husin et al. (2020a) emphasized the analysis on the crown of the tree. Significant strata was obtained using *t*-test, which was then used to develop a detection model to detect infected and healthy trees. Multiple linear regression equations were used to analyze the significant strata. It was found that the crown density profile and crown parts in a healthy oil palm tree was distinct from a BSR infected oil palm tree. Five significant strata were fitted into the prediction model using *t*-test and showed 92.5% accuracy in classifying healthy and unhealthy oil palm tree (Husin et al., 2020a).

Table 1 summarized the machine learning techniques mentioned in this article (Tee et al., 2021; Husin et al., 2020b; Mohd Hilmi Tan et al., 2021; Rehman et al., 2019; Hassan & Maji, 2022; van Dijk et al., 2021).

**Table 1:** Summary of machine learning types mentioned in this review article.

Machine learning type	Approach	Description and method	Ref.
Decision tree (DT)	Supervised regression	Determine the class of a given feature by mapping observations of data. Splits the dataset into a few tree-like models using nodes (testing attributes) and edges (for branching) by the value of selected attributes to split dataset into their respective leaves (labelling classes).	Husin et al. (2020b), Mohd Hilmi Tan et al. (2021)
Discriminant analysis (DA)	Supervised classification	Generative model. Estimate the posterior probability to classify observation into its respective class. Evaluate the dissimilarities between groups of objects with regards to several variables to assign such observation into pre-defined groups.	Husin et al. (2020b), Rehman et al. (2019)
Naïve Bayes (NB)	Supervised classification	Based on Bayes decision theorem. Classify objects to their respective class by finding the highest conditional possibility, using simple Bayesian probabilistic classifier. Assumes that the features are strongly independent against each other.	Husin et al. (2020b), Mohd Hilmi Tan et al. (2021)
Support vector machine (SVM)	Supervised classification	Separate dataset in a dimensional space into classes using a hyperplane or line. The optimal hyperplane is located and made as a decision line. Binary class separation.	Husin et al. (2020b), Mohd Hilmi Tan et al. (2021)
Nearest neighbor (NN)	Supervised classification	Stores current cases, measures similarity of new cases by distance function and classify accordingly. K-nearest neighbor (kNN) algorithm is commonly utilized for pattern recognition and multivariate analysis.	Husin et al. (2020b), Mohd Hilmi Tan et al. (2021)
Ensemble modelling (EM)	Supervised regression	Categorize new data based on prebuilt sets of classifiers. Assigns various weight to the predefined classifiers for weighted voting. Classify by using combined predictions of the classifiers. Example, Random forest (RF), which is an EM method that classify objects by taking a weighted vote from multiple decision trees.	Husin et al. (2020b), Rehman et al. (2019)
Artificial neural network (ANN)	Supervised	Imitates human brain function to complete complex tasks and make decisions. Enable computer’s learning ability by having multilayer and back-propagation. Used for determination of nonlinear combinations. Example, multilayer perceptron (MLP), which is an ANN feed-forward model. MLP maps input data to a set of appropriate outputs.	Tee et al. (2021), Mohd Hilmi Tan et al. (2021)
Convolutional neural network (CNN)	Supervised	Similar to an MLP. Consist of convolutional layers, pooling layers and fully connected layers. Convolutional layer handle feature extraction from dataset. Pooling layer down sample the dataset to reduce computational complexity. Fully connected layer connect every input with every output by weight.	Tee et al. (2021), Hassan & Maji (2022)
Generative adversarial network (GAN)	Unsupervised	Utilize supervised loss for training through discriminator and generator models. The discriminator model is able to discover and learn the patterns in input data automatically. The generator model is then able create new samples or data based on existing training dataset.	Tee et al. (2021), van Dijk et al. (2021)

In a further study, Husin et al. (2020b) attempted to classify oil palm tree into healthy and three different level of infection levels. Five features were used, namely number 13, 14, 15, 16, 17 from the top which were denoted by C650, C700, C750, C800 and C850, respectively. Six machine learning techniques were used for classification model development, namely decision tree (DT), discriminant analysis (DA), naïve Bayes (NB), support vector machine (SVM), nearest neighbor (NN) and ensemble modelling (EM). As a conclusion, kernel naïve Bayes (KNB) was found to be the most accurate model (model accuracy of 74.5%, multiple levels accuracies of 85%, Kappa coefficients of 0.80) (Husin et al., 2020b).

To understand the disease development for long-term solutions, Husin et al. (2021) used temporal laser scanning data to identify suitable time frames for monitoring the progress of the disease symptoms. Crown area and canopy strata at 850 cm from the top were identified as the most suitable parameters to be used. It was found that BSR infection can be discovered by comparing the 2 and 4-month scan images of the oil palm tree (Husin et al., 2021).

The result also demonstrated the effect of *G. Boninense* infection can be differentiated at the later stage as early as 4-month after the first inspection Therefore, the current practice of disease censuses that are done every 6 or 12 months could be shortened to a faster cycle. Since the BSR infection can kill a palm tree in 12 months, a more regular census could help avoid crop damage by allowing early identification, prevention and treatment measures (Husin et al., 2021).

Table 2 summarizes the BSR classification techniques using TLS.

**Table 2:** Summary of BSR classification techniques using TLS.

Detection tool	Method of analysis/classification	Parameter	Results	Ref.
FARO laser scanner focus 3D	Pearson correlation and linear regression	Trunk area and perimeter. Canopy size.	Perimeter of trunk, area of trunk and canopy size had significant correlation level with. BSR level.	Khairunniza-Bejo & Vong (2014)
FARO laser scanner focus 3D	Linear, two-factorial, quadratic, cubic, quartic, fifth model	Canopy strata, crown area, frond number and frond angle	Linear model showed highest accuracy in detecting healthy tree, and respectably high accuracy for healthy-unhealthy tree detection.	Husin et al. (2020c)
FARO laser scanner focus 3D	*t*-test, multiple linear regression equations	Crown density profile and significant crown strata	Five most significant strata were fitted into the prediction model and showed 92.5% classification accuracy.	Husin et al. (2020a)
FARO laser scanner focus 3D	Machine learning. DT, DA, NB, SVM, NN, EM	C200, C850, crown area, frond number and frond angle	KNB technique was found to be the model with the highest classification accuracy.	Husin et al. (2020b)
FARO laser scanner focus 3D	Kruskal–Wallis test and the Wilcoxon *post-hoc* test.	Significant strata, crown area, and frond metrics.	Tree with BSR could be detected by comparing crown strata number 17 (850 cm from top) and the crown area of the oil palm TLS image at 4-months after first inspection.	Husin et al. (2021)

#### Radio detection and ranging

Radio detection and ranging is also known as radar. Radar system transmits energy in the form of microwave signal into space, and then detect the reflected signals from the object. Similar to LiDAR, radar is also an active remotely sensed sensor (Khosrokhani, Khairunniza-Bejo & Pradhan, 2016). Radar is not impacted by distortion issues since the system is less impacted by weather conditions (Tee et al., 2021).

Toh et al. (2019) attempted to detect BSR by utilizing L band image of microwave synthetic aperture radar (SAR) satellite. Potentially, the L band SAR is able to detect difference in healthy and infected oil palm by analyzing the backscatter coefficient. The study found that BSR caused significant biophysical properties changes that can be detected by the microwave radar backscattering return (Toh et al., 2019).

Mohd Najib et al. (2020) utilized SAR that is called ALOS PALSAR 2 (Japan aerospace exploration agency (JAXA), Tokyo, Japan). Visible (red) data from landsat thematic mapper was used to obtain a full view of a plantation. This is a form of microwave remote sensing. Manipulation of horizontal-horizontal (HH) and horizontal-vertical (HV) polarizations of ALOS PALSAR data were able to distinguish oil palm trees and water bodies. Red spectra L-band from landsat data (optical) was able to detect buildings and vertical-horizontal (VGH) polarization from sentinel C-band data was able to detect clear land. Analysis of variance (ANOVA) was used to extract the threshold values in order to discriminate oil palm as compared to other plants such as rainforest and rubber plantations. The purpose of the research was to utilize ALOS PALSAR 2 to effectively map the oil palm plantation, which the research team successfully achieved an accuracy of 98.36% and 0.78% kappa coefficient. The research team reported that the total palm area in Malaysia was 3.48% higher compared to the land area described by the Malaysian palm oil board (Mohd Najib et al., 2020).

Hashim et al. (2021a) collected the ALOS PALSAR-2 dataset and classify BSR disease through multilayer perceptron (MLP) and Random forest (RF). The research also utilized imbalance approach (Sythetic minority over-sampling technique (SMOTE)) to classify BSR disease in the oil palm trees. The result demonstrated that cross-polarized backscatter (HV) polarization gave a more sensitive detection of the crop canopy structure. Both MLP and RF classifier were able to predict BSR at high accuracies (Hashim et al., 2021a).

Table 3 summarizes the BSR classification techniques using radar.

**Table 3:** Summary of oil palm and BSR research involving radar.

Detection tool	Method of analysis/classification	Parameter	Results	Ref.
SAR satellite	Microwave backscatter coefficient	L band image	BSR cased significant biophysical properties that can be detected by microwave radar backscattering return.	Toh et al. (2019)
ALOS PALSAR 2	Analysis of variance (ANOVA)	Threshold values for different land use/coverage in Malaysia	Oil palm plantation can be accurately mapped in Peninsular Malaysia (98.36%, 0.78 kappa coefficient). Total palm area was 3.48% higher than the value reported by the Malaysian palm oil board.	Mohd Najib et al. (2020)
ALOS PALSAR 2	Machine learning. MLP and RF	HV polarization of crop canopy structure	MLP and RF are capable in predicting BSR with high accuracies. Using the most significant variables, MLP had a balanced accuracy (BCR) of 95.65%. RF had a BCR of 92.70%.	Hashim et al. (2021a)

#### Tomography

Tomography produces a 2D cross-sectional view of the interior of an object by measuring ray transmission (Tee et al., 2021).

Abdullah et al. (2013) utilized GammaScorpion, which is a portable gamma-ray computed tomography system to detect BSR disease. The portable tomography system was built at the Malaysian nuclear agency of Malaysia. Despite successful system utilization, no statistical data was produced by the research team to determine the accuracy of the 2D image produced by the system (Abdullah et al., 2013). Using gamma ray can potentially poise a health risk to system operator as gamma ray emits ionizing radiation, which may cause chemical and physical damage when the energy is transferred into a living tissue (Chaturvedi & Jain, 2019).

Ishaq et al. (2014) utilized PiCUS sonic tomograph to scan the oil palm sample for visual symptoms. The team placed a set of sensors that are capable of detecting internal lesion around the sample tree trunk. By calculating the sensor distance and also the sound velocities based on the number of times the sound waves flight after knocking PiCUS electronic hammer on a placed nail, the team was able to analyze the internal damage of the tree. Using GSM, it was confirmed that the accuracy of BSR detection using PiCUS sonic tomograph was 96% (Ishaq et al., 2014).

Table 4 summarizes the BSR classification techniques using tomography.

**Table 4:** Summary of oil palm and BSR research involving tomography.

Detection tool	Method of analysis/classification	Parameter	Results	Ref.
GammaScorpion	Not provided	Not provided	Accuracy statistics not provided. Quality cross sectional image obtained.	Abdullah et al. (2013)
PiCUS sonic tomograph	Tomograph analysis	Threshold values for different land use/coverage in Malaysia	Using GSM, it was confirmed that the accuracy of BSR detection using PiCUS Sonic tomograph was 96%.	Ishaq et al. (2014)

#### Intelligent electric nose

An intelligent electric nose (e-nose) device is a technology that imitates human olfactory sensation. The device creates a composite response that is unique to each odorant (Tee et al., 2021; Khosrokhani, Khairunniza-Bejo & Pradhan, 2016).

Rahmat et al. (2020) created a disposable modified screen-printed carbon electrode (SPCE) to sense the stress induced by BSR in an oil palm tree. The modified electrode was created using reduced graphene oxide (rGO) and zinc oxide nanoparticles (ZnO-NPs) as surface modifiers. By using the differential pulse voltammetry (DPV) technique, the team was able to study the electrochemical signals collected from healthy and stress levels crude extracts. The results showed ZnO-NPs/rGO/SPCE modified electrode displayed decent sensitivity towards stressed oil palm leaves crude extracts. The modified electrode also showed decent stability and reproducibility. The results showed the potential of ZnO-NPs/rGO/SPCE to detect the stress in oil palm leaves induced by BSR (Tee et al., 2021; Rahmat et al., 2020). The modified electrode needs to be stored in low temperature to maintain the performance of the electrode (Rahmat et al., 2020).

Table 5 summarizes the BSR classification techniques using e-nose.

**Table 5:** Summary of oil palm and BSR research involving e-nose.

Detection tool	Method of analysis/classification	Parameter	Results	Ref.
ZnO-NPs/rGO/SPCE	Differential pulse voltammetry (DPV) technique	Stress induced by oil palm tree that is infected by *G. Boninense*	Modified electrode showed decent sensitivity, stability and reproducibility in the detection of stressed oil palm.	Rahmat et al. (2020)

#### Electrical properties

There is a potential to research the electrical properties of soil and leaf in order to detect BSR disease. The presence of *G. Boninense* may affect the water intake of an oil palm leading to a potential change in electrical properties (Tee et al., 2021).

Khaled et al. (2018) tested the leaf samples of oil palm tree of various BSR infection levels using electrical spectroscopy. A solid test fixture (16451B; Keysight Technologies, Hachioji, Japan) that was connected to an impedance analyzer (4294A; Agilent Technologies, Hachioji, Japan) was used. It was hypothesized that classifying BSR infection can be done by analyzing electrical properties of the leaves of oil palm tree. Impedance, capacitance, dielectric constant and dissipation factor of the leaf samples were the electrical properties utilized to determine if the parameters could be used as discriminators to classify the level of BSR infection. The method of analysis used by Khaled et al. (2018) to determine the most significant frequencies were genetic algorithm (GA), RF, and support vector machine-feature selection (SVM-FS).

The significant frequencies obtained using method of analysis mentioned above could be used to classify the features. SVM and ANN were used to test the accuracy of BSR detection. By using the selection model comparative feature analysis the highest accuracy was achieved by the significant frequencies produced by SVM-FS (overall accuracy is 88.64% with kappa coefficient of 0.8480 and low mean absolute error of 0.1652). It goes to show that SVM classifier performed better than ANN in this scenario. Among the electrical properties evaluated, impedance was shown to be the best classifier with an overall accuracies of more than 80% across different BSR infection severity level, thus showing impedance was a suitable electrical property to be utilized in the assessment of BSR infection level using spectroscopy (Khaled et al., 2018).

Hamim et al. (2019) determined that the soil electrical resistance at 15 cm around the basal stem was different between BSR infected oil palm and healthy oil palm where the mean electrical resistance of healthy oil palm trees were significantly higher compared to BSR infected trees. A new index, namely K-index was created to detect BSR infection. Combination of the mean electric resistance from eight points of measurement and its K-index gave better results in the detection of BSR. The developed model achieved an accuracy of 82%, with a 100% successful rate during validation. This research showed that soil electrical resistance could be a key discriminant to differentiate non-infected and infected trees. However, the model was only qualitative and was not tested to classify the trees based on the level of BSR infection (Hamim et al., 2019).

Aziz et al. (2021) detected *G. boninense* infection in oil palm seedlings using electrical properties. The data for soil moisture content (MC in percent), electrical conductivity (EC in S/cm), and temperature (T in °C) were collected daily using an IoT approach in which the readings from MEC10 (Dalian Endeavour Technology Co., Ltd., Dalian, China) soil sensors were transferred to the ThingSpeak cloud every hour over a 6-month monitoring period using a 3G Internet connection. The results showed that the overall mean of MC, EC, and T in both monthly and weekly analyses was lower in infected seedlings than in healthy seedlings. Furthermore, healthy seedlings were significantly different from infected seedlings in all parameters in the weekly analysis, according to a Student’s t-test at a 5% significance level. The results of this study suggested that soil MC, EC, and T could be used as indicators of *G. boninense* infection, particularly for weekly data (Aziz et al., 2021).

Table 6 summarizes the BSR classification techniques using the electrical properties of oil palm.

**Table 6:** Summary of oil palm and BSR research involving electrical properties of oil palm.

Detection tool	Method of analysis/classification	Parameter	Results	Ref.
Electrical spectroscopy	GA, RF, SVM-FS were used to detect significant frequencies. SVM and ANN used to classify BSR severity.	Impedance, capacitance, dielectric constant and dissipation factor of oil palm tree leaves.	SVM classifier performed better at classifying BSR compared to ANN, with overall accuracies of over 80%.	Khaled et al. (2018)
Soil moisture sensor	K-index	Soil electrical resistance	Able to differentiate healthy and infected oil palm at 82% accuracy.	Hamim et al. (2019)
MEC10 (Dalian Endeavour Technology Co., Ltd., Dalian, China)	T-test at a 5% significance level	Soil moisture content (MC in percent), electrical conductivity (EC in S/cm), and temperature (T in °C)	Soil MC, EC, and T could be used as indicators of *G. boninense* infection, particularly for weekly data	Aziz et al. (2021)

#### Thermal sensor

A thermal sensor is a type of sensor that captures the temperature distribution in an object. The sensor is capable of capturing thermal images without contact with the object, at the same time without destructing the object (Hashim et al., 2021b).

Khairunniza-Bejo et al. (2018) used thermal images of canopy regions of healthy and infected BSR oil palm trees to differentiate the healthy and infected palm trees. The images were processed and statistically analyzed to generate intensity values that correlate to the thermal characteristics of the plants. To minimize the input’s dimensionality, principal component analysis (PCA) was employed. Principal components one and three were utilized in k-nearest neighbor (kNN) and support vector machine (SVM) classifier. When compared to kNN, the SVM-based model achieved a higher classification accuracy of 89.2% for the training set and 84.4% for the test set (Khairunniza-Bejo et al., 2018).

Johari et al. (2021) used similar method to extract significant features for dataset in detecting infected BSR oil palm seedlings. Several classification models were later used namely linear discriminant analysis (LDA), quadratic discriminant analysis (QDA), SVM and kNN. The SVM (fine Gaussian) classification produced the best results, with an accuracy of 80% (Johari et al., 2021).

Hashim et al. (2021b) aimed to use thermal imaging to classify infected and non-infected trees. ANOVA was used as part of the method of statistical analysis to assess temperature characteristic of infected and non-infected oil palm trees. To overcome the issues with different sample sizes, imbalance data approaches such as random undersampling (RUS), random oversampling (ROS) and synthetic minority oversampling (SMOTE) were used in this study. The result showed that maximum temperature (Tmax) of oil palm trunk and combination feature maximum and minimum temperature of oil palm trunk had a higher correct classification for the healthy and infected oil palm trees for the ROS-Random forest (ROS-RF) and had a robust success rate, with 87.10% correct classification for healthy and 100% for infected oil palm trees (Hashim et al., 2021b).

Table 7 summarizes the BSR classification techniques using thermal sensors.

**Table 7:** Summary of oil palm and BSR research involving thermal sensors.

Detection tool	Method of analysis/classification	Parameter	Results	Ref.
FLIR E60 thermal camera (FLIR Systems, Inc., Wilsonville, OR, USA)	Principal component analysis (PCA), SVM and kNN	Pixel values of thermal images of oil palm tree	When compared to kNN, the SVM-based model achieved a higher classification accuracy of 89.2% for the training set and 84.4% for the test set	Khairunniza-Bejo et al. (2018)
FLIR E60 thermal camera (FLIR Systems, Inc., Wilsonville, OR, USA)	Linear discriminant analysis (lda), quadratic discriminant analysis (QDA), SVM and kNN	Pixel values of thermal images of oil palm tree seedlings	The SVM (fine Gaussian) classification produced the best results, with an accuracy of 80%.	Johari et al. (2021)
FLIR T620 IR infrared thermal imaging camera (FLIR Systems, Inc., Wilsonville, OR, USA)	ANOVA (Statistical Analysis). RUS, ROS, SMOTE (Imbalance Approach). Machine learning method used for classification: NB, MLP, RF	Temperature values of thermal images of oil palm tree	Soil MC, EC, and T can be used as indicators of *G. boninense* infection, particularly for weekly data	Hashim et al. (2021b)

#### Hyperspectral sensor

Hyperspectral sensors provide images that contain high spectral information and limited spatial information (Sara et al., 2021). Data collected by such sensors consist of hundreds or thousands of continuous spectral bands to track the spectral responses of the object over a continuous wavelength. As a result, detailed spectral signatures are obtained to identify the object (Tee et al., 2021; Khosrokhani, Khairunniza-Bejo & Pradhan, 2016).

Hyperspectral images can be captured using hyperspectral sensors that are either ground-based, airborne or space-borne (Tee et al., 2021). From the articles reviewed in this article, most hyperspectral sensors are ground-based or airborne.

Liaghat et al. (2014) utilized reflectance spectroscopy to classify BSR into three stages of BSR infection levels. The study team utilized a portable hyperspectral ASD field spectroradiometer (by FieldSpec® HandHeld; Analytical Spectral Devices Inc., Boulder, CO, USA) to obtain visible-near infrared (VIS-NIR) spectral reflectance. The team utilized LDA, quadratic discriminant analysis (QDA), kNN and NB classifiers to classify and distinguish the spectra of infected trees, and found that the spectra of infected trees’ leaves could be detected among healthy tree leaves even when the tree was mildly infected. The research team were able to achieve 97% accuracy without false negatives using kNN based classification model (Liaghat et al., 2014).

Ahmadi et al. (2017) measured the VIS-NIR spectral reflectance data using a ground based and portable spectroradiometer GER model 1500 (Geophysical and Environmental Research Corporation, Millbrook, NY, USA). Using ANN analysis technique on raw, first, and second derivative spectroradiometer datasets, the team were able to discriminate and classify BSR severity even at the initial stage of infection. It was found that the highest accuracy occurred in the green wavelength, which is within visible range. By using first derivative spectral data, accuracy of classification for healthy and infected trees were 83.3% (at 540 nm) and 100% (at 550 nm), respectively. The analysis showed that the sensitive frond number modelled by ANN provided 100% accuracy for frond number nine compared to frond number 17, which proved ANN was capable of classifying BSR at early stage of infection with respectable accuracies (Ahmadi et al., 2017).

Ahmadi et al. (2022) further studied on the use of an UAV together with ANN model to analyze and detect early stage BSR infection in an oil palm tree. UAV that Ahmadi et al. (2022) used was a Hexacopter Tarot 680PRO folding vehicle TL68P00. The image collection was done using Canon Powershot SX260 HS digital camera that was NIR-modified using an external NIR filter. The research team confirmed that a combination of green and NIR spectral band showed the best performance in the identification of BSR infected tree at the early stage. Using ANN, classification of healthy palms was accurate while the classification of early infected oil palm was significantly lower. The training model reports detection of healthy oil palm at 99.27% accuracy while it is 88.50% accuracy for early infected oil palm. For corresponding classes, the accuracy dropped to 75% for healthy oil palm and 57.14% for early infected oil palm. This scenario was due to half of the early infected oil palm were misclassified as healthy oil palm since early infected oil palm shows little differences at areas such as chlorophyll content at early stages of infection (Ahmadi et al., 2022).

According to Noor Azmi et al. (2020), by analyzing the relationship between VIS reflectance, NIR reflectance and BSR infection levels, non-infected trees tend to show lower VIS reflectance and higher NIR reflectance. Whereas infected trees tend to show dissimilar spectral patterns which is dependent on the physiological state and the morphology of the leaves (Noor Azmi et al., 2020).

Noor Azmi et al. (2020) studied the possibility of detecting early *G. Boninense* infection when the plant shows no BSR infection symptom by using VIS-NIR hyperspectral images. The sample size consisted of 28 oil palm seedlings that are five months old. Fifteen seedlings were inoculated from the total sample with *G. Boninense*. Five months later, the spectral reflectance of fronds 1 (F1) and 2 (F2) of the oil palms were obtained using the VIS-NIR hyperspectral images captured by FireflEYE S185 (Cubert GmbH, Ulm, Germany) camera. The wavelength of the hyperspectral camera was from 450 to 950 nm (124 bands) that covered VIS and NIR regions with a sampling interval of 4 nm. By identifying the high separation between non-inoculated and inoculated seedlings, significant bands were then obtained. It was found that by using reflectance data of F1 and SVM kernel, classification accuracy between non-inoculated and inoculated seedlings were at 100% by using 35 and 18 NIR bands. In contrast, using F2 or a combination of F1 and F2 (F12), the highest classification accuracy was only 93% (Noor Azmi et al., 2020).

Azmi et al. (2021) also further studied on the topic of asymptomatic BSR disease identification using hyperspectral data analyzed by several machine learning models. Using the finding in previous study where it showed significant differences between non-inoculated seedlings and inoculated seedlings in the NIR spectrum. A total of 23 machine learning models were developed which include DT, DA, logistic regression, NB, SVM, kNN, EM together with various types of kernels. The performance of the models was evaluated using F-score, where it is a measure of sensitivity and precision. The research found that coarse Gaussian SVM with nine bands was the best model as it combines excellent accuracy, use of small number of bands and shorter performance time. Coarse Gaussian SVM scored 95.21% in F-score (Azmi et al., 2021).

Khairunniza-Bejo et al. (2021) investigated the possibility of using fewer wavelengths between one and five in order to counter the economic aspect of hardware design. The results were then compared to detection results obtained from vegetation indices developed using spectral reflectance extracted from two wavelengths from the same hyperspectral sensor. In both single-band reflectance and vegetation index datasets, the results showed that a kernel with a simple linear separation between two classes would be more suitable for *G. boninense* detection than the others. A linear SVM with a single-band reflectance at 934 nm was identified as the best detection model because it was not only cost effective but also demonstrated high accuracy (94.8%), specificity (92.5%) and sensitivity (97.6%) (Khairunniza-Bejo et al., 2021).

Lee et al. (2022) utilized an UAV in combination with MLP to learn the spectral features from oil palm tree of different BSR infection level. The UAV was deployed to capture red, green, and blue (RGB) image and hyperspectral images (captured by the hyperspectral camera mounted on the same UAV). The hyperspectral image sample collected consist of two healthy, five slightly infected, five moderately infected and three severely infected oil palm trees. The machine learning model was trained with ground truth, which consisted data reported by trained surveyors. It was discovered that MLP model showed the highest overall accuracy which was 86.67% compared to SVM and one dimensional CNN, which only achieved 66.67% and 73.33%, respectively (Lee et al., 2022).

Kurihara et al. (2022) used an UAV with hyperspectral imager and used RF machine learning algorithm to classify oil palm disease into various infection severity category. The hyperspectral imager is a sequential two-dimensional imager (Genesia Corp., Tokyo, Japan). By using a tunable filter, the 2-D imager was capable of changing the spectral bands sequentially. The UAV used was a DJI Agriculture Series drone. By pre-processing the image, reflectance-based hyperspectral cube was created for each scene that was captured by the imager. Using the reflectance spectra of individual oil palm tree, the team utilized concentric disk segmentation to segmentize the tree crown. The team was able to calculate the mean reflectance spectra of each segment of the hyperspectral cube of the scene. The results showed that only some spectral bands in the red-edge region could be used adequately to classify BSR infection levels (Kurihara et al., 2022). This was also explained by Horler, Dockray & Barber (1983), where the red-edge region were tightly linked to the light absorption by the chlorophylls on the plant cells. Therefore, this parameter reflected the abundance of chlorophyll, which healthy plants typically have a richer chlorophyll content compared to stressed or diseased plants (Horler, Dockray & Barber, 1983).

Table 8 summarizes the BSR classification techniques using hyperspectral sensors.

**Table 8:** Summary of Oil Palm and BSR research involving hyperspectral sensors.

Detection tool	Method of analysis/classification	Parameter	Results	Ref.
Hyperspectral ASD field spectroradiometer (FieldSpec® HandHeld)	LDA, QDA, kNN and NB	VIS-NIR spectral reflectance data	kNN classification model shows 97% accuracy in BSR infection level classification without false negatives.	Liaghat et al. (2014)
Portable spectroradiometer GER model 1500	ANN	VIS-NIR spectral reflectance data	The sensitive frond number modelled by ANN provided 100.0% accuracy for frond number 9 compared to frond 17.	Ahmadi et al. (2017)
UAV (Hexacopter Tarot 680PRO folding vehicle TL68P00) and NIR modified Canon Powershot SX260 HS	ANN	Mean and standard deviation values from G, R and NIR bands that was found to be the most representative, adjusted for best circle radius and threshold limit	The training model reports detection of healthy oil palm at 99.27% accuracy while it is 88.50% accuracy for early infected oil palm. For corresponding classes, the accuracy dropped to 75% for healthy oil palm and 57.14% for early infected oil palm.	Ahmadi et al. (2022)
FireflEYE S185 camera (Cubert GmbH)	SVM	Spectral reflectance of fronds 1 (F1) and 2 (F2) of the leaflets from oil palm seedlings	Using reflectance data of F1 and SVM kernel, classification accuracy between non-inoculated and inoculated seedlings were at 100% by using 35 and 18 NIR bands. In contrast, using F2 or a combination of F1 and F2 (F12), the highest classification accuracy was only 93%.	Noor Azmi et al. (2020)
FireflEYE S185 camera (Cubert GmbH)	DT, DA, logistic regression, NB, SVM, kNN, EM	Spectral reflectance	Coarse Gaussian SVM scored 95.21% in F-score. Coarse Gaussian SVM with nine bands was the best model as it combines excellent accuracy, use of small amount of bands and shorter performance time.	Azmi et al. (2021)
Not applicable. Uses secondary data from Noor Azmi et al. (2020)	Linear SVM	Band reflectance with wavelength between one and five as compared to spectral reflectance from hyperspectral sensor.	A linear SVM with a single-band reflectance at 934 nm was identified as the best detection model because it was not only cost effective but also demonstrated high accuracy (94.8%), specificity (92.5%) and sensitivity (97.6%).	Khairunniza-Bejo et al. (2021)
UAV	MLP, SVM, CNN	Spectral bands of hyperspectral images	MLP model showed the highest overall accuracy which is 86.67% compared to SVM and 1 dimensional CNN, which only achieved 66.67% and 73.33% respectively.	Lee et al. (2022)
DJI agriculture series drone with sequential two-dimensional imager (Genesia Corp., Tokyo, Japan)	RF	Concentric disk segmentation and mean reflectance spectra	Only some spectral bands in the red-edge region could be used adequately to classify BSR infection levels.	Kurihara et al. (2022)

#### Multispectral sensor

Multispectral sensors provide images with little spectral information and high spatial information (Sara et al., 2021). The sensor is capable of capturing the energy that is reflected or emitted from a specific object or area in multiple discrete bands of the electromagnetic spectrum (Tee et al., 2021).

Santoso, Tani & Wang (2017) utilized the high resolution and synoptic overview capability of QuickBird satellite to map and detect BSR disease. QuickBird offers multispectral images that contains pixel values of all bands. Santoso, Tani & Wang (2017) attempted to evaluate the machine-learning models to predict BSR disease in plantations and map the BSR infection distribution. Three machine learning models were used, namely SVM, RF and classification and regression tree (CART) model. Through the study, it was found that RF model performed the best in the prediction and classification of BSR infection in the plantation. This is determined using several matrices such as overall accuracy, producer accuracy, user accuracy and kappa value. The team trained the model using 75% of total data available and the remaining 25% of the data were used for testing. RF classifier model achieved 91% overall accuracy. RF model was also capable of distinguishing infected and non-infected oil palm within the plantation (Santoso, Tani & Wang, 2017).

Santoso et al. (2018) attempted to utilize WorldView-3 imagery and supervised machine learning algorithm to classify the severity of BSR infection. WorldView-3 is a high-resolution commercial satellite. The research team tested the eight multispectral bands available on WorldView-3 and their ability to identify four levels of BSR infection. Due to the high rate of photosynthesis, non-infected trees tend to absorb light in the spectrum ranges from band one to five. That phenomenon explains why healthy trees showed low reflectance values towards spectrum range one to five. Reflectance values for band seven and eight, which are the near infrared bands are higher for the non-infected trees and lower for the infected trees. This is due to the healthy oil palm do not use near infrared bands for metabolism, leading to high reflectance values (Santoso et al., 2018). This also explained why other studies such as Liaghat et al. (2014) utilized near infrared bands to differentiate healthy and BSR infected oil palms. Band 6 (red edge band) was commonly not used since healthy and severely unhealthy oil palm both showed similar reflectance value, which proved that the reflectance value using band 6 was not a good differentiator. In terms of machine learning algorithm, for this study SVM model showed higher accuracy to detect BSR as compared to RF and DT (overall accuracy range for DT, RF and SVM was between 47.5% and 51.7%). It was also noted that the higher the number of observations in the training set, the accuracy of learners besides SVM model showed a drop in accuracy.

Anuar et al. (2020) utilized Parrot Sequoia multispectral camera system that was mounted on a DJI Phantom Matrice, which is a lightweight quadcopter-type UAV. The team found that it was incredibly difficult to detect early infected trees among the non-infected trees, since the tree shows little detectable symptoms during early stage of infection. The research team utilized object-based image analysis (OBIA) to classify oil palm to different BSR severity levels. In terms of accuracy, a combination of edge-based segmentation and merge algorithm using full-lambda schedule (FLS), SVM and three-band data of green, red and NIR showed the best accuracy at 91.8%. When the data of individual band were further analyzed, the accuracies were more modest, between 65.5% to 76.2% range. The research team found that by combining several bands together, better classification accuracies were obtained (70% to 90%). This result showed that OBIA was a reliable tool to analyze multispectral images in order to identify moderate and severely infected oil palm trees. Detection of early BSR infection may be possible if other algorithms and classifiers that are more advanced were used to analyze multispectral and hyperspectral aerial images (Anuar et al., 2020).

Table 9 summarizes the BSR classification techniques using multispectral sensors.

**Table 9:** Summary of oil palm and BSR research involving multispectral sensors.

Detection tool	Method of analysis/classification	Parameter	Results	Ref.
QuickBird satellite	SVM, RF and classification and regression tree (CART) model	Mean pixel values derived from the four bands of QuickBird imagery	RF model was the best at predicting, classifying, and mapping BSR infection RF model’s overall accuracy was 91%.	Santoso, Tani & Wang (2017)
WorldView-3	DT, RF and SVM	Mean pixel values from the eight bands of WorldView-3	SVM model showed higher accuracy to detect BSR as compared to RF and CART (overall accuracy range for DT, RF and SVM was between 47.5% and 51.7%).	Santoso et al. (2018)
Parrot Sequoia multispectral camera system, mounted on a DJI phantom matrice (UAV)	OBIA. Edge-based segmentation and merge algorithm using FLS, SVM	Three-band data of green, red and NIR	By combining several bands together, better classification accuracies were obtained (70% to 90%). OBIA is a good tool to analyze multispectral images for moderate and severe levels of BSR.	Anuar et al. (2020)

## Summary of literature review

Oil palm is a key agricultural resource in many developing nations, especially Indonesia and Malaysia. Oil palm also contributes to one-third of the world’s vegetable and fat supply, which 85% of the world’s supply come from Indonesia and Malaysia (Bharudin et al., 2022; Bentivoglio, Finco & Bucci, 2018). However, a necrotrophic fungus namely *G. Boninense* is causing a severe basal stem rot (BSR) infection. *G. Boninense* restrict the water uptake by the oil palm trees, which are the host, and infected oil palm tree potentially lose about 50% to 80% yield and the oil palm tree may be dead within 2 to 3 years (Siddiqui et al., 2021; Bharudin et al., 2022; Nurazah et al., 2021).

BSR infection causes an estimated economic loss of RM 225 million to 2.2 billion *per annum*. BSR can potentially destroy 860,610 hectares worth of oil palm plantations by year 2040 (Bharudin et al., 2022; Rani et al., 2020; Olaniyi & Szulczyk, 2020).

Many approaches to manage BSR were discussed in chapter II of this review article, which include physical, chemical and biocontrol. Physical control is expensive as it requires huge labor and manpower, which with the shortage of labor it is even more difficult to physically control the spread of BSR (Siddiqui et al., 2021; Rani et al., 2020; Ho et al., 2016). Chemical and biocontrol methods often have biosafety concerns, and most methods are proven inadequate to significantly control the spread of BSR (Siddiqui et al., 2021; Nurazah et al., 2021). Genetic modification methods were also discussed, but the solution is far from being implemented on a commercial scale. The solution to controlling the spread of BSR is by the means of early detection, isolation and sanitization (Siddiqui et al., 2021; Hushiarian, Yusof & Dutse, 2013).

However, detecting BSR at early infection stage proves to be a challenge as oil palm that was infected by *G. Boninense* showed little to no symptoms. Therefore, the solution to early detection of BSR should be portable, affordable, high tolerance towards environmental conditions, low power consumption and most importantly able to detect early stage BSR infection at high accuracy (Rani et al., 2020).

Detecting BSR can be done using the manual method, which is *via* the manual labor and visual inspection. However, at early stage of BSR infection, the symptoms are often not visible thus making such solution unreliable. Besides, visual inspection of oil palm tree for BSR infection often requires huge amount of experience and expertise, which the skillset is often not transferable. It will take very long period of time for the skillset to be passed down, therefore making manual inspection unfavorable (Siddiqui et al., 2021; Alam, Er & Begum, 2015; Tee et al., 2021; Ibrahim et al., 2020).

Lab methods were also discussed, namely GSM, PCR and ELISA-PABS. GSM is not advisable for use on commercial scale due to biosafety concerns. Accuracy over GSM also raises some concerns. PCR is considered a gold standard test due to the test’s high accuracy and sensitivity. However, PCR test requires expensive infrastructure, highly skilled manpower to conduct such tests, complex testing protocols and high reagent costs. PCR also do not support on-site testing for the same reason. Besides, PCR do not classify the level and status of infection for the tested sample (Siddiqui et al., 2021; Hushiarian, Yusof & Dutse, 2013; Tee et al., 2021; Martinelli et al., 2014). PCR is also susceptible to sample contamination (Siddiqui et al., 2021; Hushiarian, Yusof & Dutse, 2013). ELISA-PABS showed better detection performance against GSM, however the test is not suitable for large scale monitoring and detection since the test is still sophisticated and time-consuming (Tee et al., 2021; Madihah, Idris & Rafidah, 2014).

Using digital data collection method seems to be a great option, since once an automated data collection and analysis method is established, the system has great potential to reduce labor hours and cost compared to manual and lab methods (Tee et al., 2021). However, accuracy of BSR has to be at least comparable, if not better than existing detection methods. Besides, the digital method has to detect BSR during early stages of infection in order for the system to be effective as a disease control measure.

TLS method was discussed in detail and the summary can be found in Table 2. All the studies discussed used FARO TLS system, but used several analytical methods while analyzing different areas of the oil palm, which include the canopy, crown, trunk and *etc*. The parameters studied were also varied. However it was worth noting, by using t-test and multiple linear regression equations to analyze the crown density profile, the five most significant strata were able to be fitted into the prediction model that produced 92.5% accuracy in disease classification. Comparing methods of machine learning, KNB was found to be the best in classifying BSR based on C200, C850, crown area, frond number and frond angle parameters (Husin et al., 2020c; Khairunniza-Bejo & Vong, 2014; Husin et al., 2020a, 2020b, 2021).

RADAR data collection method was summarized in Table 3. Studies showed that radar technology was able to detect the biophysical properties that were caused by BSR. Radar was also used to more accurately map the total plantation area as compared to the area reported by the Malaysian Palm Oil Board. It was shown that by utilizing ALOS PALSAR 2 to collect HV polarization of crop canopy structure, analyzed with machine learning methods, balanced accuracy in BSR disease prediction was 95.65% and 92.70%, for MLP and RF, respectively (Toh et al., 2019; Mohd Najib et al., 2020; Hashim et al., 2021a).

The studies related to tomography was summarized in Table 4. It was noted that the accuracy statistic using tomography system was not disclosed with the use of GammaScorpion. The use of PiCUS sonic tomograph reported a BSR detection accuracy of 96% (Abdullah et al., 2013; Ishaq et al., 2014). However, there was health concerns over the use of gamma-ray system such as GammaScorpion due to long term gamma ray emission, since gamma ray is an ionizing radiation (Tee et al., 2021). The setup of PiCUS sonic tomograph, despite being highly accurate, was unable to provide a commercial scale BSR disease monitoring due to the complex setup (Ishaq et al., 2014).

Study related to the usage of e-nose that was linked to BSR was summarized in Table 5. It was promising to find out that by using ZnO-NPs/rGO/SPCE and DPV technique to analyze the stress levels crude extracts, the modified electrode demonstrated decent sensitivity towards stressed oil palm. The test also demonstrated good stability and reproducibility. However, the study did not provide any accuracy data for reference. It was also noted that the modified electrode needs to be stored in low temperatures to maintain the performance of the electrode (Rahmat et al., 2020).

The use of electrical properties of oil palm and soil was researched and the summary of the findings can be found in Table 6. Using electrospectroscopy, studying several parameters such as the impedance, capacitance, dielectric constant and dissipation factor of oil palm tree leaves, SVM was able to classify BSR infection better compared to ANN, with overall accuracies of a respectable 80% and above. Soil electrical resistance could also be used to classify healthy and impacted oil palm tree. By using a soil moisture sensor and analyzed using K-index, differentiation accuracy (healthy and unhealthy oil palm) of 82% was achieved (Khaled et al., 2018; Hamim et al., 2019).

Table 7 summarizes the summary of studies that utilized thermal sensors to detect and classify oil palm infected with BSR. By using the pixel values and the temperature values provided by the thermal images, the combination of image analysis together with machine learning methods such as SVM provided BSR detection accuracy from 80% to 100% for infected palm trees (Hashim et al., 2021b; Khairunniza-Bejo et al., 2018; Johari et al., 2021).

With the improvement and availability of UAV, options were opened for the use of hyperspectral sensors as the sensors could now be attached to an UAV. The researchers were able to analyze oil palm tree from an entirely different dimension. The summary of articles using hyperspectral sensors is in Table 8. Several combinations were used to analyze the oil palm tree to classify the disease severity, the most significant findings were using the combination of hyperspectral sensors and machine learning to analyze the VIS-NIR spectral reflectance. ANN method reported a 100.0% accuracy, kNN classification model reported a 97% accuracy with no false negatives (Liaghat et al., 2014; Ahmadi et al., 2017). It was also noteworthy that by using ANN to evaluate the most representative mean and standard deviation values from G, R and NIR bands, adjusted for the best circle radius and threshold limit, 88.50% accuracy was achieved in detecting oil palm at the early infection stage. Several controlled studies were done on seedlings to study the possibility of detecting BSR even when an oil palm did not show any BSR related symptoms. Using SVM kernel, the accuracy achieved was from 93% to 100% (Noor Azmi et al., 2020).

Research using multispectral sensors were reviewed and the summary can be found in Table 9. Multispectral sensors give high spatial information. Using six vegetation indices derived from VIS-NIR data provided by QuickBird Satellite only gave 59% to 67% accuracy. However, using machine learning such as SVM and RF to analyze the mean pixel values from the four bands of QuickBird imagery, the accuracy improved significantly. RF method reported a 91% overall accuracy when 75% of the data were used to train the model and the rest of the data were used for testing purpose. However, analyzing the mean pixel values of WorldView-3 satellite was not promising, even when machine learning models were used, as accuracy range was from 47.5 % to 51.7% (Santoso, Tani & Wang, 2017). Using Parrot Sequoia together with UAV, analyzing three-band data of green, red and NIR using OBIA gave an accuracy of 70% to 90%, which could be viewed as promising (Anuar et al., 2020).

## Potentials and limitations of deep learning in the analysis of digital data

As an overview, among all the digital data collection methods, hyperspectral sensors using UAV seems to be a more reliable way as using the hyperspectral images together with machine learning typically give rise to the highest overall accuracy and performance. Other digital methods such as TLS and handheld hyperspectral sensor may be accurate but those methods are impractical for monitoring on a commercial scale. With recent advancement in UAV and machine learning, making analyzing oil palm and classifying BSR severity level using UAV more reliable. There is still space to improve the accuracy of BSR infection detection, especially during the early infection stage, as the oil palm may show little symptoms throughout the plant. The previous articles covered multiple machine learning techniques such as DT, DA, NN, NB, EM (RF), SVM and ANN, while only one article covered the use of convolution neural network (CNN) as the potential solution to detect and classify BSR disease (Lee et al., 2022). Using one dimensional CNN, the accuracy reported was from 66.67% to 73.33%. However, there is space for improvement since CNN is often considered as an upgraded version of ANN (Tee et al., 2021).

Other plant disease studies, such as Xiao et al. (2020) showed that strawberry leaves images that were analyzed using CNN models reported strawberry diseases classification accuracy of at least 98%. Sharma, Berwal & Ghai (2020) compared the application of CNN on full images (F-CNN) or segmented image (S-CNN) of tomato leaves, and it was found that S-CNN model demonstrated much higher accuracy compared to F-CNN model. The accuracy of S-CNN model was 98.6%.

However, the use of CNN typically require a large dataset, which is not common for analysis on plant disease (Bi & Hu, 2020). In the case where the number of model parameters exceed the number of dataset, a small training dataset would give rise to data overfitting problem, which is a problem where the model responded too tightly to a training dataset which made the model unable to adapt to additional data and be used for reliable testing (Tee et al., 2021). When sample size is limited, the accuracy of a CNN model will be significantly decreased. Nguyen et al. (2021) showed that by having only 40 images, the accuracy achieved by 2D-CNN and 3D-CNN models were only from 71% to 75% during the detection of grapevine viral disease (Nguyen et al., 2021).

To overcome the issue with limited dataset, data augmentation can be considered. Data augmentation increases the number of labelled images by means of rotation or even adjustment in brightness, contrast and sharpness. This method can be used to increase the number of labelled image available for training and testing. Data augmentation is able to reduce the impact of limited training dataset problem, but this method is unable to reproduce practical diversity, which is why generative adversarial network (GAN) may be a better solution in case of limited dataset (Bi & Hu, 2020).

GAN is able to increase the diversity of the data by generating new datasets for the training of the model. GAN model consists of two sub-networks, namely a generator and a discriminator. The generator captures the training data distribution. The discriminator estimates the probability that an image is from the training dataset rather than being generated from the generator (Bi & Hu, 2020). Bi & Hu (2020) displayed that by using pure CNN, the test accuracy was only around 60%. Using the proposed method, which is by having CNN together with classic data augmentation, Wasserstein Generative Adversarial Network (with the Wasserstein distance and gradient penalty that are used in the loss function) and label smoothing regularization (WGAN-GP-LSR), the accuracy of testing was increased to 84.78% (Bi & Hu, 2020).

## Conclusion and knowledge gap

UAV together with hyperspectral sensor technology have shown great promises to monitor BSR that is caused by *G. Boninense*. Deep learning such as CNN and GAN machine learning methods were rarely used on oil palm tree data and through the other plant disease studies, deep learning showed great potentials in improving existing BSR detection methods. By improving the overall accuracies of BSR detection, the possibilities of detecting BSR infection at an early stage will also potentially improve, making development of a reliable system combining both remote sensors and deep learning in the early detection of BSR on a commercial scale possible in the near future.

## Additional Information and Declarations

### Data Availability

The following information was supplied regarding data availability: This research article is a literature review and does not have any raw data or code.
